# The dosimetric effect of mixed‐energy IMRT plans for prostate cancer

**DOI:** 10.1120/jacmp.v12i4.3563

**Published:** 2011-11-15

**Authors:** Jong Min Park, Chang Heon Choi, Sung Whan Ha, Sung‐Joon Ye

**Affiliations:** ^1^ Department of Radiation Applied Life Science Seoul National University Graduate School Seoul; ^2^ Institute of Radiation Medicine, Medical Research Center Seoul National University College of Medicine Seoul; ^3^ Department of Radiation Oncology Seoul National University College of Medicine Seoul; ^4^ Department of Intelligent Convergence Systems Seoul National University Seoul Republic of Korea

**Keywords:** intensity‐modulated radiation therapy, mixed‐energy plans, prostate cancer, radiation treatment planning, dose‐volumetric analysis

## Abstract

We investigated the effect of mixing high‐ and low‐energy photon beams on the quality of intensity‐modulated radiation therapy (IMRT) plans for patients with prostate cancer. Three different plans for each of twenty patients were generated using either 6 MV or 15 MV alone, and both 6 and 15 MV beams. All the planning parameters, goals, and constraints were set to be identical except beam energy. The dose distributions were similar in terms of target coverage, conformity, and homogeneity regardless of beam energy. The $V_{70\text{Gy}}$ of rectal wall in 6 MV, 15 MV and mixed‐energy plans was 16.7%, 17.9%, and 16.3%, respectively, while $V_{40\text{Gy}}$ was 55.6%, 53.2%, and 50%. The mean dose to femoral heads in 6 MV, 15 MV, and mixed‐energy plans were 31.7 Gy, 26.3 Gy, and 26.2 Gy, respectively. The integral dose of 6 MV plans was 7% larger than those of 15 MV or mixed‐energy plans. These results indicated that mixed‐energy IMRT plans could take advantage of the dosimetric characteristics of low‐ and high‐energy beams. Even though the reduction of dose to the organs at risk may not be clinically relevant, mixing energy in an IMRT plan for deep‐seated tumors can improve the overall plan quality.

PACS number: 87.55.ne

## I. INTRODUCTION

Intensity‐modulated radiation therapy (IMRT) can deliver the conformal dose distributions by varying radiation intensities within each field according to the fluence maps (i.e., intensity maps) optimized by a treatment planning system (TPS). The conformal dose distributions are often stipulated by dose and dose‐volume constraints for targets and organs at risk (OARs).^(^ ^1^ ^)^ By virtue of this capability, IMRT has enabled the delivery of conformal doses to the target while sparing the surrounding normal tissues, which can furthermore allow dose escalation — which is beneficial for the outcomes of radiotherapy.^(^ ^2^ ^–^ ^5^ ^)^

The selection of photon energy has been an issue in the conventional three‐dimensional conformal radiotherapy (3D CRT). Photon beams of low energy (≤6MV) have been used to treat superficial tumors located within a short distance from skin surface because of their limited penetrating power. On the contrary, photon beams of high energy (>6 Mv) have advantage in penetrating power and skin sparing, and thus have been used to treat deep‐seated tumors. It has been shown that the narrow penumbra of low‐energy beams results in tighter dose distributions around a target, minimizing irradiation of nearby OARs, although the regions near beam entry ports receive higher doses.^(^ ^6^ ^)^ On the other hand, high‐energy beams have negative aspects such as increasingly diffused beam boundaries due to the long lateral range of secondary electrons^(^ ^7^ ^)^ and generation of secondary neutrons. The secondary neutrons are generated from the interactions between photons and treatment head when using 10 MV or higher, due to the photoneutron effect.^(^ ^8^ ^)^ Since secondary neutrons contribute to the unnecessary irradiation of a patient, they should be avoided.

In radiotherapy for prostate cancer, high‐energy beams are traditionally used for 3D CRT, since the target is located deep inside the body. However the majority of IMRTs for prostate cancer delivered today are with lower energy (6–10 MV) because of no neutron production in a linear accelerator treatment head.^(^ ^8^ ^)^ Furthermore, a number of studies demonstrated that there was no clear benefit in use of higher photon energy in IMRT even for deep‐seated tumors such as prostate cancer.^(^ ^9^ ^–^ ^15^ ^)^ In those studies, the energy becomes less important as the number of IMRT beams increases. The IMRT plans of low energy are clinically equivalent with those of high energy in terms of target coverage, homogeneity, conformity, and OAR savings if a sufficient number of fields are used (usually nine fields or more).^(^ ^10^ ^,^ ^16^ ^)^ Benefits of low energy include minimizing the head leakage, internal scatter, and concern from secondary neutrons. However, IMRT with low energy deposits high doses in the region peripheral to the target, and generally requires a more complex plan containing a greater number of fields, beam segments, and MU.^(^ ^12^ ^)^ This increases treatment delivery times, integral doses, and irradiation of surrounding organs. Adverse skin reactions are also a concern for low‐energy treatment of deep‐seated targets, particularly in large patients. However, some studies have demonstrated that the skin dose differences between using low‐ and high‐energy photon beams for prostate cancer are not significant if a sufficient number of fields are used.^(^ ^16^ ^,^ ^17^ ^)^ Although some differences between using low‐ and high‐energy photon beams depend on the dose calculation method employed,^(^ ^18^ ^)^ using low‐energy photon beams in IMRT is the preference today due to the previously mentioned benefits.

There is a wide variety of literature on selection of beam energy and other IMRT parameters, such as the number of fields and beam orientations for prostate treatment.^(^ ^9^ ^–^ ^13^ ^,^ ^15^ ^–^ ^17^ ^,^ ^19^ ^–^ ^21^ ^)^ Findings and recommendations were sometimes inconsistent, even providing some conflicting conclusions. The previous studies about IMRT energy selection were performed by comparing IMRT plans with either low‐ or high‐energy alone, but none of them have addressed the potentials of mixed‐energy IMRT plans. Since the cross section of pelvis is oval in shape, a penetration depth of beam path is quite different depending on gantry angles. Thus, we investigated the effect of changing beam energy according to the penetration depths on the quality of IMRT plans for prostate cancer. In this study, higher energy was chosen for fields with deeper penetration depths, while lower energy was selected with shallower depths. The comparisons between mixed‐energy plans and single‐energy plans of either low or high energy were made while keeping the beam arrangement, the number of beams, and the weight of dose constraints identical for all plans.

## II. MATERIALS AND METHODS

### A. Patient population

From February 2008 to August 2010, twenty patients who underwent radiotherapy alone for prostate cancer were the subjects of this study (IRB Number: H‐1103‐088‐356).

### B. Simulation and contouring

All patients underwent a computed tomography (CT) simulation in a supine position. CT images were acquired with a slice thickness of 2 mm. The primary planning target volume $\left({\mathrm{PTV}}_{P}\right)$ was defined with a margin of 2 cm around the prostate and seminal vesicles in all directions except the posterior and inferior directions, where a margin of 1 cm was added. The boost PTV $\left({\mathrm{PTV}}_{B}\right)$ was defined with a margin of 1 cm around the prostate in all directions except the posterior direction, where a margin of 0.7 cm was added. The rectum wall, bladder, femoral heads, and urethra were contoured as OARs based on the CT images. Instead of rectum, the rectum wall subtracted by air cavity or feces was defined as an OAR in our institution (as has been done in other institutions).^(^ ^22^ ^)^ The rectum wall was segmented from the level of ischial tuberosities to the rectosigmoid flexure per the Radiation Therapy Oncology Group (RTOG) protocol. The bladder and femoral heads were delineated based on the CT images. Most of patients underwent the scanning procedure with a full bladder.

### C. Planning

**Figure 1:**
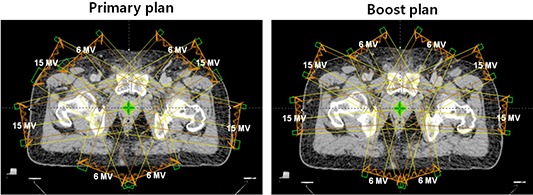
Beam orientations are shown for primary and boost plan of mixed‐energy. Half of the beams which had to penetrate deeper depths were 15 MV photon beams, while the others were 6 MV photon beams.

IMRT plans were generated retrospectively using the Eclipse system (version 8.6) with 6 and 15 MV photon beams of Varian 21EX (Varian Medical Systems, Palo Alto, CA). For IMRT, the sliding window technique using a 120 leaf millennium multileaf collimator was used. The anisotropic analytical algorithm (AAA) was used for dose calculations at a grid of 2.5 mm. The total prescription dose was 81 Gy with a daily dose of 1.8 Gy. Since this was a comparative study, the same prescription was applied to all plans. The prescription dose of the primary plans was 50.4 Gy to ${\text{PTV}}_{P}$, and the prescription dose of the boost plans was 30.6 Gy to ${\text{PTV}}_{B}$. A primary plan for ${\text{PTV}}_{P}$ and a separate boost plan for ${\text{PTV}}_{B}$ were generated using either 6 MV or 15 MV alone and mixed‐energy of 6 and 15 MV for each patient. We set the plan goals for PTVs such that 95% of the prescribed dose covered at least 95% of the PTV, and the PTV volume receiving >103% of the prescription was limited to zero. However, it was necessary to apply more stringent dose limits to the real planning process than those described above,^(^ ^19^ ^,^ ^20^ ^)^ since the optimization algorithm could not satisfy all the demands placed on it, and segmentation degraded the plans. To achieve these objectives, a constraint for $D_{100\%}$ was set to receive 99.5% of the prescription and a constraint for the maximum dose $\left(D_{\max}\right)$ was set to receive 102% of the prescription in the optimization process for the primary plans. For the boost plans, a constraint for $D_{100\%}$ was set to receive 102% of the prescription and a constraint for the maximum dose $\left(D_{\max}\right)$ was set to receive 103% of the prescription in the optimization process. The initial optimization constraints and weightings are summarized in Tables 1 and 2. Initially these constraints and weightings were the same for all plans regardless of beam energy, and then only dose volume constraints were modified during the optimization process by either relaxing or tightening the real‐time updated dose‐volume histograms (DVHs) to accommodate the patient‐specific differences in the structures of interest. This approach might result in biased results rather than fair comparisons. Since the process of an IMRT optimization is a compromise between PTV goals and OAR constraints, the excessive OAR constraints generally ruin the PTV coverage or uniformity. Therefore, the OAR dose‐volume constraints only were modified unless the PTV coverage and uniformity were ruined, and the weightings were kept the same for 6 MV, 15 MV, and mixed‐energy plans during the optimization. A 2 cm wide ring structure (as a pseudo target) that is 7 mm apart from PTV was used to achieve high‐dose gradients around PTV. The normal tissue objectives were used to avoid hot spots in the normal tissues and to obtain sharp dose gradients around PTV. This controls doses outside the target structure (body subtracted by PTV). The priority of normal tissue was 120 and the distance from target border was set to 1 cm. The start and the end dose for normal tissue objective were set to 105% and 60%, and the falloff was set to 0.05. The IMRT doses were delivered to the ${\text{PTV}}_{P}$ using eight coplanar and nonopposed beams at gantry angles of 160°, 100°, 60°, 40°, 320°, 300°, 260°, and 200°. For ${\text{PTV}}_{B}$, eight coplanar beams were used at gantry angles of 165°, 95°, 65°, 30°, 330°, 295°, 265°, and 195°. These gantry angles are used in our clinic for IMRT plans for prostate cancer. For the primary plan of mixed‐energy, 15 MV photon beams at the gantry angles of 100°, 60°, 300°, and 260° were used since they penetrated the longest four beam paths, while 6 MV were used for the rest of the gantry angles. For the boost plan of mixed‐energy, 15 MV at the gantry angles of 95°, 65°, 295°, and 265° were used, while 6 MV were used at the rest of the gantry angles in order to use 15 MV photon beams at the fields penetrating the four longest beam paths. We mixed high‐ and low‐energy photon beams equally in a plan. This is shown in Fig. 1. A fluence map for each beam was generated at the end of IMRT optimization. The MLC leaf sequence was then calculated to deliver the fluence map with a series of MLC segments at a fixed‐dose rate of 600 MU/min. All IMRT plans were normalized such that 95% of the prescription dose covered at least 95% of the PTV after the optimization process. The primary and boost plan were combined to generate a sum (composite) plan. The planning was done by a single physicist and the clinical aspects were reviewed by a single oncologist.

**Table 1:** The initial dose‐volume constraints for primary plan.

*Structure*	*Initial Dose‐volume Constraints*	*Relative Priority*
${\text{PTV}}_{P}$ ^a^	$D_{100\%}$ ^b^ ≥99.5% of the prescription dose	150
	$D_{98\%}\geq 100\%$ of the prescription dose	150
	$D_{2\%}\leq 101\%$ of the prescription dose	150
	$D_{\max}$ ^c^ ≤102% of the prescription dose	150
Rectum Wall	$V_{50\text{Gy}}$ ^d^ ≤0%	100
	$V_{44\text{Gy}}\;\leq 20\%$	100
	$V_{41\text{Gy}}\;\leq 25\%$	100
	$V_{30\text{Gy}}\;\leq 50\%$	100
Bladder	$V_{50\text{Gy}}\;\leq 0\%$	100
	$V_{44\text{Gy}}\;\leq 30\%$	100
	$V_{41\text{Gy}}\;\leq 40\%$	100
	$V_{30\text{Gy}}\;\leq 55\%$	100
Femoral Heads	$V_{34\text{Gy}}\;\leq 0\%$	80
	$V_{30\text{Gy}}\;\leq 10\%$	80
	$V_{23\text{Gy}}\;\leq 50\%$	80
Body	$D_{\max}\leq 51.4\text{Gy}$	180

a ${\text{PTV}}_{P}$ is primary planning target volume.

b $D_{n\%}$ means the dose received by the n% volume of the target volume.

c $D_{\max}$ means the maximum dose received.

d $V_{\text{nGy}}$ means the percentage volume irradiated by n Gy or more of a certain structure.

**Table 2:** The initial dose‐volume constraints for boost plan.

*Structure*	*Initial Dose‐volume Constraints*	*Relative Priority*
${\text{PTV}}_{B}$ ^a^	$D_{100\%}$ ^b^ ≥102% of the prescription dose	150
	$D_{98\%}\geq 102.2\%$ of the prescription dose	150
	$D_{2\%}\leq 102.8\%$ of the prescription dose	150
	$D_{\max}$ ^c^ ≤103% of the prescription dose	150
Rectum Wall	$V_{30\text{Gy}}$ ^d^ ≤0%	100
	$V_{26\text{Gy}}\;\leq 20\%$	100
	$V_{19\text{Gy}}\;\leq 50\%$	100
Bladder	$V_{30\text{Gy}}\;\leq 0\%$	100
	$V_{26\text{Gy}}\;\leq 30\%$	100
	$V_{19\text{Gy}}\;\leq 55\%$	100
Femoral Heads	$V_{16\text{Gy}}\;\leq 0\%$	80
	$V_{15\text{Gy}}\;\leq 20\%$	80
	$V_{12\text{Gy}}\;\leq 50\%$	80
Body	$D_{\max}\leq 31.5\text{Gy}$	180

a ${\text{PTV}}_{B}$ is boost planning target volume.

b $D_{n\%}$ means the dose received by the n% volume of the target volume.

c $D_{\max}$ means the maximum dose received.

d $V_{\text{nGy}}$ means the percentage volume irradiated by n Gy or more of a certain structure.

### D. Dose‐volumetric analysis

Dose‐volumetric analysis was performed by using DVHs of the treatment plans of individual patients. The homogeneity index (HI) and the conformity index (CI) were calculated for the ${\text{PTV}}_{P}$ and ${\text{PTV}}_{B}$ from the primary and the boost plans, respectively. The HI evaluates the dose homogeneity within the PTV.^(^ ^23^ ^)^ The HI was calculated as follows: $D_{5\%}/D_{95\%}$, where $D_{n\%}$ is the minimum dose delivered to n% of the PTV. The CI was calculated as follows: (volume within the 95% isodose) / (volume of the PTV). The values of CI or HI close to unity indicate greater conformity or homogeneity. The gradient measure (GM) defined in Eclipse was calculated from both primary and boost plans. The gradient measure is an indication of dose gradients, which is the difference in centimeters between the equivalent sphere radii of the prescription and half prescription isodoses. A smaller GM indicates higher dose gradients around the target.

The maximum and mean dose to ${\text{PTV}}_{P}$ and ${\text{PTV}}_{B}$ were calculated from the primary, boost, and sum plans. To evaluate irradiated volumes of the rectum wall and bladder, the volumes that received 70 Gy, 66.6 Gy, 50 Gy, 40 Gy, and $20\;\text{Gy}(V_{70\text{Gy}},V_{66.6\text{Gy}},V_{50\text{Gy}},V_{40},\;\text{and}\;V_{20\text{Gy}})$ were calculated from the sum plans. The mean dose and $D_{50\%}$ were also calculated from the sum plans. For the femoral heads, the maximum and mean doses $V_{50\text{Gy}}$, $V_{45\text{Gy}}$, $V_{30\text{Gy}}$ and $D_{50\%}$ were calculated from the sum plans. For the body, $V_{24.5\text{Gy}}$ (cc) was calculated from the sum plans to evaluate the volume which received about 30% of the prescription dose. The mean doses, $D_{\mathrm{mean}}$, of bladder, rectum, and femoral heads in the primary and boost plans were compared separately, as well. The average DVHs for each OAR were also generated from the individual DVHs. A paired t‐test was used to calculate the statistical difference of dose‐volumetric results between 6 MV and 15 MV plans, 6 MV and mixed‐energy plans, and 15 MV and mixed‐energy plans.

## III. RESULTS

The dose‐volumetric results of all plans are shown in Table 3.

**Table 3:** Dose‐volumetric comparison of 6 MV, 15 MV, and mixed‐energy IMRT.

*Variable*	*6 MV IMRT*	*15 MV IMRT*	*Mixed‐energy IMRT*	*p‐value (6 vs. 15)* ^*a*^	*p‐value (6 vs. mixed)* ^*b*^	*p‐value (15 vs. mixed)* ^*c*^
*Primary Plan* (mean±standard deviation)						
Conformity Index	1.04±0.04	1.05±0.02	1.04±0.02	0.902	0.281	<0.001
Homogeneity Index	1.05±0.01	1.06±0.01	1.06±0.01	0.407	<0.001	<0.001
Gradient Measure (cm)	2.62±0.29	2.29±0.20	2.34±0.24	<0.001	<0.001	*0.006*
Max Dose to ${\text{PTV}}_{P}$ (Gy)^d^	51.58±0.77	51.39±0.38	51.93±0.48	0.231	*0.034*	<0.001
Mean Dose to ${\text{PTV}}_{P}$ (Gy)	49.31±0.47	49.33±0.67	49.69±0.27	0.907	<0.001	*0.037*
Mean Dose to Rectum Wall (Gy)	33.81±2.87	32.72±3.88	32.13±3.91	*0.039*	*0.002*	0.100
Mean Dose to Bladder (Gy)	29.21±8.64	29.71±8.62	29.12±8.85	0.082	0.685	*0.003*
Mean Dose to Femoral Heads (Gy)	22.27±3.89	18.61±3.35	18.23±3.12	<0.001	<0.001	0.414
*Boost Plan* (mean±standard deviation)						
Conformity Index	1.08±0.03	1.10±0.03	1.09±0.03	<0.001	0.075	*0.041*
Homogeneity Index	1.04±0.01	1.06±0.01	1.06±0.01	<0.001	<0.001	0.512
Gradient Measure (cm)	1.79±0.27	1.67±0.18	1.71±0.18	<0.001	*0.013*	*0.015*
Max Dose to ${\text{PTV}}_{B}$ (Gy)^e^	30.81±0.38	30.95±0.27	31.21±0.33	0.051	<0.001	<0.001
Mean Dose to ${\text{PTV}}_{B}$ (Gy)	29.83±0.26	30.14±0.19	30.22±0.22	<0.001	<0.001	*0.044*
Mean Dose to Rectum Wall (Gy)	12.68±1.88	12.33±1.92	11.71±2.19	0.169	*0.003*	*0.011*
Mean Dose to Bladder (Gy)	9.63±4.15	9.77±4.52	9.53±4.52	0.198	0.323	<0.001
Mean Dose to Femoral Heads (Gy)	9.38±3.07	7.79±2.37	7.93±2.24	<0.001	<0.001	0.199
*Sum Plan* (mean±standard deviation)						
Maximum Dose (Gy)	81.63±0.99	82.22±2.28	82.27±0.62	0.332	<0.001	0.925
Mean Dose to ${\text{PTV}}_{P}$ (Gy)	71.47±2.53	72.04±2.62	72.01±2.63	<0.001	<0.001	0.413
Mean Dose to ${\text{PTV}}_{B}$ (Gy)	79.18±0.70	79.78±0.38	80.05±0.46	<0.001	<0.001	<0.001
Mean Dose to Rectum Wall (Gy)	46.50±3.44	45.06±4.88	43.84±4.83	*0.050*	<0.001	*0.015*
$V_{70\text{Gy}}$ ^f^ of Rectum Wall (%)	16.7±5.4	17.9±6.3	16.3±6.1	*0.002*	0.222	<0.001
$V_{66.6\text{Gy}}$ of Rectum Wall (%)	19.2±5.5	20.6±6.7	18.7±6.5	*0.003*	0.332	<0.001
$V_{50\text{Gy}}$ of Rectum Wall (%)	38.6±7.7	39.5±8.7	37.0±8.3	0.151	*0.012*	<0.001
$V_{40\text{Gy}}$ of Rectum Wall (%)	55.6±9.4	53.2±9.9	50.0±10.3	0.240	*0.012*	*0.002*
$V_{20\text{Gy}}$ of Rectum Wall (%)	95.6±4.4	89.4±8.4	89.0±8.2	*0.001*	<0.001	0.675
$D_{50\%}$ ^g^ of Rectum Wall (Gy)	42.69±5.56	42.31±6.76	39.90±6.80	0.708	*0.006*	<0.001
Mean Dose to Bladder (Gy)	38.30±13.45	39.45±12.76	38.65±12.96	*0.043*	0.532	<0.001
$V_{70\text{Gy}}$ of Bladder (%)	19.9±10.3	21.1±11.6	20.5±11.5	*0.003*	0.068	*0.001*
$V_{66.6\text{Gy}}$ of Bladder (%)	23.4±11.8	24.4±12.9	23.7±12.8	*0.015*	0.381	<0.001
$V_{50\text{Gy}}$ of Bladder (%)	39.6±17.3	41.0±17.7	39.8±17.7	<0.001	0.538	<0.001
$V_{40\text{Gy}}$ of Bladder (%)	47.4±19.4	47.5±19.1	47.5±19.6	0.968	0.962	0.974
$V_{20\text{Gy}}$ of Bladder (%)	65.6±21.7	65.9±20.5	64.3±21.5	0.718	0.117	*0.008*
$D_{50\%}$ of Bladder (Gy)	35.57±19.53	37.52±20.19	35.72±20.47	*0.002*	0.725	<0.001
Max dose to Femoral Heads (Gy)	55.22±8.93	48.01±6.58	47.50±6.73	<0.001	<0.001	0.432
Mean Dose to Femoral Heads (Gy)	31.67±6.53	26.32±5.36	26.16±4.92	<0.001	<0.001	0.728
$V_{50\text{Gy}}$ of Femoral Heads (%)	4.9±8.8	0.6±1.6	0.4±1.1	*0.019*	*0.022*	0.186
$V_{45\text{Gy}}$ of Femoral Heads (%)	9.5±12.7	2.4±5.3	1.7±4.1	*0.002*	*0.002*	*0.049*
$V_{30\text{Gy}}$ of Femoral Heads (%)	58.9±24.9	32.4±20.8	31.8±19.9	<0.001	<0.001	0.799
$D_{50\%}$ of Femoral Heads (Gy)	31.77±5.94	25.86±5.30	25.86±4.86	<0.001	<0.001	0.994
$V_{24.3\text{Gy}}$ of Body (cc)	2,324±507	2,059±466	2,125±482	<0.001	<0.001	<0.001
Integral Dose ($10^{5}$ Gy‐cc)	1.18±0.26	1.10±0.24	1.10±0.25	<0.001	<0.001	0.110
Average MU^h^	952±124	871±80	966±61	*0.001*	0.551	<0.001

a *P*‐value (6 vs. 15) means p‐value from the comparison of the results between 6 MV IMRT and 15 MV IMRT plans.

b *P*‐value (6 vs. Mixed) means p‐value from the comparison of the results between 6 MV IMRT and mixed‐energy IMRT plans.

c *P*‐value (15 vs. Mixed) means p‐value from the comparison of the results between 15 MV IMRT and mixed‐energy IMRT plans.

d ${\text{PTV}}_{P}$ means the primary planning target volume.

e ${\text{PTV}}_{B}$ means the boost planning target volume.

f $V_{\text{nGy}}$ means the percentage volume irradiated by n Gy or more of a certain structure.

g $D_{n\%}$ means the dose received by the n% volume of the target volume.

h MU is monitoring unit.

### A. Conformity and homogeneity of the target

There were no clear differences in the conformity and homogeneity index among 6 MV, 15 MV, and mixed‐energy plans. However, boost plans of 6 MV beams showed a slightly better target dose conformity and homogeneity than boost plans of 15 MV beams (1.08 vs. 1.10 for CI and 1.04 vs. 1.06 for HI, p<0.001 and p<0.001, respectively). Both of the primary and boost plans of 6 MV beams showed slightly better target dose homogeneity than those of mixed‐energy (1.05 vs. 1.06 in primary plans and 1.04 vs. 1.06 in boost plans, p<0.001 and p<0.001, respectively). And both of the primary and boost plans of mixed‐energy beams showed slightly better target dose conformity than those of 15 MV beams (1.04 vs. 1.05 in primary plans and 1.09 vs. 1.1 in boost plans, p<0.001 and p=0.041, respectively). There was no difference in homogeneity index between 15 MV and mixed‐energy plans.

### B. Maximal and mean dose to target

No clear differences were observed among 6 MV, 15 MV, and mixed‐energy plans. The mean doses to ${\text{PTV}}_{P}$ and ${\text{PTV}}_{B}$ in 6 MV sum plans were slightly lower than the others with statistical significance but the difference was not large. The differences of maximal and mean dose to ${\text{PTV}}_{P}$ between 15 MV and mixed‐energy plans were negligible and statistical significances were not reached. The mean dose to ${\text{PTV}}_{B}$ was slightly higher in mixed‐energy sum plans than those in 15 MV sum plans, but differences were less than 1% of the prescription dose (80.1 Gy vs. 79.8 Gy, p<0.001).

### C. Dose to rectum wall

The rectal wall volume of 15 MV plans that received 70 Gy and 66.6 Gy were larger than those of 6 MV plans (17.9% vs. 16.7% for $V_{70\text{Gy}}$ and 20.6% vs. 19.2% for $V_{66.6\text{Gy}}$, p=0.002 and p=0.003, respectively). The average value of $V_{20\text{Gy}}$ of 15 MV plans was smaller than that of 6 MV plans (89.4% vs. 95.6% for $V_{20\text{Gy}}$, p=0.001). The statistical significances of differences in $V_{50\text{Gy}}$ and $V_{40\text{Gy}}$ were not reached. The mixed‐energy plans always showed lower dose to rectum wall than 6 MV and 15 MV plans. The differences of $V_{70\text{Gy}}$, $V_{66.6\text{Gy}}$, and $V_{50\text{Gy}}$ between 6 MV and mixed‐energy plans were relatively small, and the statistical significances of $V_{70\text{Gy}}$ and $V_{66.6\text{Gy}}$ were not reached. However, the differences of $V_{40\text{Gy}}$ and $V_{20\text{Gy}}$ were relatively large with statistical significances (55.6% vs. 50% for $V_{40\text{Gy}}$ and 95.6% vs. 89% for $V_{20\text{Gy}}$, p=0.012 and p<0.001, respectively). The mean doses and $D_{50\%}$ were larger in 6 MV and 15 MV plans than in mixed‐energy plans with statistical significances (46.5 Gy in 6 MV and 45.1 Gy in 15 MV vs. 43.8 Gy in mixed‐energy for mean dose, and 42.7 Gy in 6 MV and 42.3 Gy in 15 MV vs. 39.9 Gy in mixed‐energy for $D_{50\%}$).

### D. Dose to bladder

Doses to the bladder of 15 MV plans were always slightly higher than those of 6 MV plans, but no significant differences were observed. The average value of $D_{50\%}$ of 6 MV plans was lower than that of 15 MV plans (35.6 Gy vs. 37.5 Gy, p=0.002) Doses to the bladder of mixed‐energy plans were always slightly higher than that of 6 MV plans. However, the differences were negligible and showed no statistical significances. Doses to bladder of mixed‐energy plans were always slightly lower than those of 15 MV plans with statistical significances even though the differences were not large.

### E. Dose to femoral heads

Doses to the femoral heads of 6 MV plans were always higher than those of 15 MV and mixed‐energy plans with statistical significances of differences. The mean doses were 31.7 Gy in 6 MV plans, 26.3 Gy in 15 MV plans (p<0.001 between 6 MV vs. 15 MV), and 26.2 Gy in mixed‐energy plans (p<0.001 between 6 MV vs. mixed‐energy). The values of $D_{50\%}$ were 31.8 Gy in 6 MV plans, 25.9 Gy in 15 MV plans (p<0.001 between 6 MV vs. 15 MV), and 25.9 Gy in mixed‐energy plans (p<0.001 between 6 MV vs. mixed‐energy). Doses to femoral heads of 15 MV plans were always higher than those of mixed‐energy plans. However statistical significances were not reached, except for $V_{45\text{Gy}}$ (p=0.049). The mean doses and $D_{50\%}$ between 15 MV and mixed‐energy plans didn't show noticeable differences and statistical significances were not reached for either of them.

### F. Dose to normal tissue and monitor unit

It was shown that the value of gradient measure in 6 MV plans was higher for both primary and boost plans than that of 15 MV and mixed‐energy plans with statistical significances of differences. The volume of body which received 30% of the prescription dose was reduced to 2,059 cc in 15 MV and 2,125 cc in mixed‐energy plans compared to 2,324 cc in 6 MV plans (p<0.001 for 6 MV vs. 15 MV and p<0.001 for 6 MV vs. mixed‐energy). Integral doses of 6 MV, 15 MV, and mixed‐energy plans were 117,655 Gy‐cc, 109,553 Gy‐cc, and 110, 148 Gy‐cc, respectively. The average value of monitor unit of mixed‐energy plans was higher than that of 15 MV and 6 MV plans, but the statistical significances of differences were not reached (952 MU in 6 MV, 871 MU in 15 MV, and 966 MV in mixed‐energy plans). It was shown that the value of gradient measure in mixed‐energy plans was higher for both primary and boost plans than that of 15 MV plans (2.34 cm vs. 2.29 cm in primary plans and 1.71 cm vs. 1.67 cm in boost plans, p=0.006 and p=0.015, respectively).

### G. Average dose‐volume histograms

**Figure 2:**
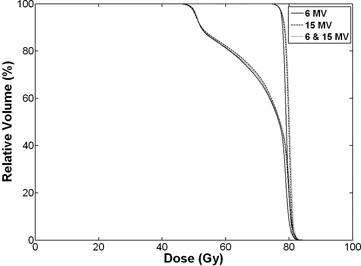
Dose volume histograms (DVHs) for the primary and boost planning target volumes (PTVs) from sum plans. The solid lines indicate DVHs of intensity‐modulated radiotherapy (IMRT) with 6 MV photon beams. The one which receives higher dose is DVH for the boost PTV and the other is DVH for the primary PTV. Dashed lines and dotted lines indicate DVHs from 15 MV and mixed‐energy plans, respectively. No clear differences are observed among the three types of plans.

**Figure 3:**
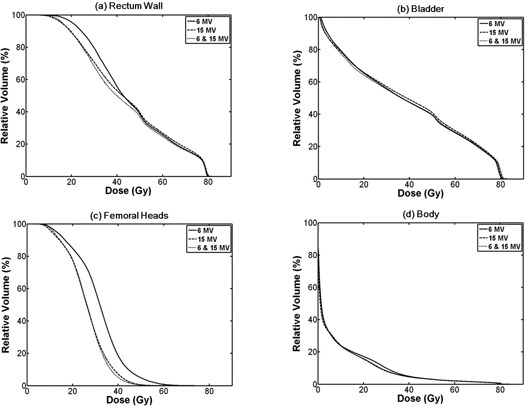
Dose volume histograms (DVHs) for organs at risk (OARs) from sum plans: (a) DVH for rectum wall; IMRT with 15 MV spares more rectum wall in high‐dose regions while 6 MV saves more in low‐dose regions; IMRT with mixed‐energy photons delivers lower dose to rectum wall through all regions than the others; (b) DVH for bladder with no clear differences among three types of plans; (c) DVH for femoral heads shows that IMRT with 6 MV delivers higher dose to femoral heads than the others; (d) DVH for body shows that IMRT with 6 MV delivers higher dose in the region from 10 Gy to 40 Gy. The solid lines indicate DVHs of intensity‐modulated radiotherapy (IMRT) with 6 MV photon beams, while dashed lines and dotted lines indicate DVHs of IMRT with 15 MV and mixed‐energy photons, respectively.

The average DVHs for primary and boost PTVs from sum plans of 6 MV, 15 MV, and mixed‐energy beams are shown in Fig. 2. The average DVHs for (a) rectum wall, (b) bladder, (c) femoral head, and (d) body from sum plans are shown in Fig. 3.

## IV. DISCUSSION

Similarly to the previous studies,^(^ ^9^ ^,^ ^10^ ^,^ ^13^ ^,^ ^24^ ^,^ ^25^ ^)^ no distinctive differences in clinical dosimetric quality between 6 and 15 MV plans were found in this study. However, in order to maintain the same levels of target coverage, conformity, and homogeneity, 6 MV plans spared more rectum wall in high‐dose regions than 15 MV, while 15 MV plans spared more rectum wall in low‐dose regions than 6 M V, even though both of them were clinically acceptable. This result was explained by the fact that low‐energy photon beams can generate tighter dose distributions around a target, and high‐energy photon beams provide better penetrating power. The higher femoral head doses in 6 MV plans than those in 15 MV plans can be understood in the same context. Since the femoral heads are located relatively far away from the prostate, they received more dosage when 6 MV photon beams were used than when 15 MV photon beams were used, due to differences in penetrating power. For the same reason, the irradiated volume of low dose and the integral dose in 6 MV plans were larger than those in 15 MV photons. The monitor units of 6 MV plans were 1.1 times higher (on average) than those of 15 MV plans.

As mentioned earlier, the previous studies have demonstrated that the selection of energy is not sensitive in the quality of IMRT plans for prostate cancer when a sufficient number of fields are used, even for exceptionally large patients.^(^ ^9^ ^,^ ^10^ ^,^ ^13^ ^,^ ^24^ ^)^ Those studies were focused on the cases of OAR doses above 50% of the prescription dose. In this study, we showed that using high‐energy beams in prostate IMRT was beneficial for saving some OARs even in low‐dose regions under 50% of the prescription dose. But there was a trade‐off of losing benefits in high‐dose regions, even though it would not be clinically significant.

Some studies have demonstrated that the absolute lifetime risk of fatal secondary malignancy due to IMRT with 15 MV photon beams, including neutron dose increases, slightly compared with that of IMRT with 6 MV photon beams (3.4% of lifetime risk for 15 MV IMRT plans while 2.9% for 6 MV IMRT plans).^(^ ^26^ ^,^ ^27^ ^)^ However, the portion of high‐energy photon beams in mixed‐energy plans was 50% in this study. A simple arithmetic estimation of the secondary cancer risk from neutrons by using mixed‐energy photon beams results in half of the risk when using 15 MV photon beams alone.

There are concerns about a wide lateral fall‐off of high‐energy photon beams due to a long lateral range of secondary electrons. This can induce an adverse affect on the aimed delivery of modulated beams.^(^ ^14^ ^)^ This characteristic of high‐energy photon beams degrades saving OARs that are located close to the target, such as the rectum and bladder. Our findings agreed with the previous studies mentioned above. However, in mixed‐energy plans, the rectum wall received consistently lower doses. Furthermore, the integral doses in mixed‐energy plans were reduced to, on average, 93% of those in 6 MV photon plans. This would be beneficial for reducing the secondary malignancy risks induced by radiotherapy.^(^ ^27^ ^)^

## V. CONCLUSIONS

The results of this study indicate that an IMRT plan with mixed‐energy photon beams is able to take advantage of both low‐ and high‐energy photon beams. Even though the reduction of dose to OARs and normal tissues may not be clinically relevant, it is worthwhile to note that mixing energy in an IMRT plan for deep‐seated tumors (such as prostate cancer) can improve the overall plan quality.
